# Multifunctional Adjuvants Affect Sulfonylureas with Synthetic Auxin Mixture in Weed and Maize Grain Yield

**DOI:** 10.3390/plants13111480

**Published:** 2024-05-27

**Authors:** Robert Idziak, Hubert Waligóra, Leszek Majchrzak, Piotr Szulc

**Affiliations:** Department of Agronomy, Poznan University of Life Sciences, Dojazd 11, 60-632 Poznan, Poland; robert.idziak@up.poznan.pl (R.I.); hubert.waligora@up.poznan.pl (H.W.); leszek.majchrzak@up.poznan.pl (L.M.)

**Keywords:** ALS herbicide, multicomponent adjuvant, maize, synthetic auxin, weed

## Abstract

A field study in the years 2017–2019 was carried out to evaluate the impact of novel adjuvant formulations on the efficacy of sulfonylurea and synthetic auxin herbicides. Treatments included nicosulfuron + rimsulfuron + dicamba (N+R+D) at full and reduced rates with three multicomponent (TEST-1, TEST-2, TEST-3) as well as standard (MSO, S) adjuvants. In this greenhouse study, *Echinochloa crus-galli* seeds were planted and treated with N+R+D at 2–3 leaf stages. The water with the desired pH (4, 7, and 9) for the preparation of the spray liquid was prepared by incorporating citric acid or K_3_PO_4_ to either lower or raise the pH of the water. Adjuvant TEST-1 added to the spray liquid at pH 4 increased the effectiveness to 68%, TEST-2 to 81%, and TEST-3 to 80%, compared to 73% and 66% with the MSO and S. The efficacy of N+R+D at pH 7 with TEST-1 increased to 83%, TEST-2 to 82%, and TEST-3 to 77%, but with MSO, it increased to 81%, and 71% with S. Adjuvants TEST-1, TEST-2, and TEST-3 in the liquid at pH 9 increased efficacy to 76 and 80%, compared to 79 and 63% with MSO or S adjuvants. N+R+D applied with TEST-1, TEST-2, and TEST-3 provided greater weed control than herbicides with surfactant (S) and similar or even better than with standard methylated seed oil (MSO) adjuvants. Maize grain yield after herbicide-with-tested-adjuvant application was higher than from an untreated check, and comparable to yield from herbicide-with-MSO treatment, but higher than from S treatment.

## 1. Introduction

Maize is characterised by poor competitiveness against weeds and their presence in maize can lead to up to 70% reduction in grain yield [1,2,3]. An effective weed control is the correct selection of an active substance appropriate to the species composition of the community, and preferably mixtures of substances with different mechanisms of action [4,5]. The herbicide efficacy depends on many factors including the selection of the active substance, environmental factors, and the quality of the spray liquid, mainly pH, hardness, and temperature of the water [6,7]. Sulfonylurea herbicides are more effective at higher and less effective at lower pH levels due to their lower solubility [8,9]. Nicosulfuron is a selective, systemic, absorbed by foliage and roots, herbicide that inhibits plant amino acid synthesis—acetohydroxyacid synthase (AHAS). Nicosulfuron and rimsulfuron ionise in aqueous solutions. The higher pH, the more intense the ionisation process, and the solubility of sulfonylurea derivatives increases [6]. This substance is stable at pH 7 and 9 but degrades quickly in acid conditions [10]. Nicosulfuron exhibits exceptional efficacy at low concentrations; nevertheless, its residues may persist in soil and, through the leaching process, may infiltrate groundwater, affecting sensitive plant species during crop rotations, resulting in phytotoxicity [11].

It is advisable in plant protection to use solutions allow maximum effectiveness to be achieved with the least possible environmental impact [12]; therefore, it makes sense to reduce the loss of active substances as much as possible, so that the lowest possible dose of the product can be applied, the greatest possible proportion of which will reach the site of action in the plant. In order to maintain the high efficacy of reduced herbicide doses, it is deliberate to add adjuvants to the spray liquid [13]. According to [14], adequacy of a pesticide can be increased by using different adjuvants, and it can also decrease quantity of an herbicide and total costs for weed management by using an adjuvant, and this means any substance in an herbicide formulation or added to the spray tank to improve herbicidal activity or application characteristics [15]. Their action, especially of multicomponent adjuvants, is very broad and they influence better mixing of herbicides; reduce spray application problems; increase droplet coverage, spray retention, and cuticle penetration; and enhance and improve an herbicide’s efficacy. Finally, adjuvants decrease the amount of herbicide applied, and lower costs for weed control [16]. Studies [17,18] indicated that an adjuvant changed the spray liquid properties but that improvements varied with the adjuvant class; therefore, the selection of the appropriate class of adjuvants can improve effectiveness of pesticides. Activating adjuvants typically increase herbicide activity; herbicide spread, retention, and absorption into plant tissues; and rain fastness, and decrease the photodegradation of the herbicide. This group includes surfactants, oils, wetting agents, certain mineral compounds, and multicomponent adjuvants [19].

Some pesticide active substances can have negative effects on non-target organisms [20,21]. Even adjuvants may be even more harmful than herbicide or fungicide formulations [22,23]; therefore, research into more environmentally friendly adjuvants should be carried out [24,25]. Some of them, e.g., surfactants, can have an unpredictable influence on human health and the ecosystem [20,21]. The activity of single-component adjuvants is incomplete but combining several ingredients in a single formulation, it is possible to obtain a product with a much broader, more comprehensive effect, so-called multipurpose adjuvants [26]. Due to the presence of many active substances, the mode of action of multipurpose adjuvants is exceptionally broad. They have the potential to reduce spray drift, decrease droplet surface tension, enhance the retention and deposition on the plant surface, slow down the evaporation of spray droplets, enhance herbicide uptake to leaves and cells, and enhance the efficacy of an herbicide. They also reduce the risk of herbicide wash-off by rain shortly after application [27,28,29].

The aim of this greenhouse study was to evaluate the effect of newly developed formulations of multifunctional adjuvants on the performance of the active substances under conditions of varying pH of the water used to prepare the spray liquid. In field trials, the effect of adjuvants on the herbicidal efficacy and phytotoxicity to crop plants of nicosulfuron with rimsulfuron and dicamba was evaluated. The effects of adjuvants and herbicides on maize grain yield and parameters were also evaluated.

## 2. Results

### 2.1. Greenhouse Experiment

The efficacy of N+R+D prepared in the spray liquid at pH 4, 7, and 9, applied at FR, was 48 and 56%; at RR, it was 45, 37, and 42% (Table 1). The addition of TEST-1 at pH 4 increased the efficacy to 68%, and TEST-2 and TEST-3 to 80–81%, compared to 73% and 66% with MSO and S. The efficacy of N+R+D at pH 7 with TEST-1 increased to 83% and TEST-2 to 82%, and 77% with TEST-3, 81% with MSO, and 71% with S. Adjuvants TEST-1, TEST-2, and TEST-3 at pH 9 increased N+R+D efficacy to 76–80%, compared to 79 and 63% with MSO and S. The average efficacy of N+R+D at pH 4 was 66%; at pH 7 and 9, it was 68%. There was a favourable effect of TEST adjuvants on N+R+D efficacy; it was higher than the application of N+R+D at RR without and with S and at a similar level to MSO.

### 2.2. Field Experiment

Weed flora of experimental fields consisted of *Chenopodium album* L. (CHEAL), *Geranium pusillum* L. (GERPU), *Fallopia convolvulus* (L.) Á. (POLCO), *Viola arvensis* L. (VIOAR), and *Echinochloa crus-galli* (L.) P. Beauv. (ECHCG). In 2017, 2018, and 2019, the total mass of weeds was 7305, 2394, and 2760 g m^–2^. The Ss coefficient characterizing the similarity of the weed communities for 2017–2018 was 35%; for 2018–2019, it was 48%; and for 2017–2019, it was 38%. The N+R+D at FR controlled CHEAL during the study years by 81–86%; at RR, it was 60–66% (Table 2). The efficacy of N+R+D with TEST adjuvants led to an increase in efficacy above the FR of herbicides to 96% (TEST-2), 93% (TEST-1), and 90% (TEST-3) in 2018, and 99–100% in 2019. N+R+D with TEST adjuvants controlled CHEAL better or equal to N+R+D with MSO and better than with S. In 2017, 95–100% ECHCG efficacy was recorded, and in 2018, 81 and 61% were recorded with FR and RR of N+R+D, and 98% with TEST-2, 99–100% with TEST-1 and TEST-3, 99% with MSO, and 93% with S. Similar results were obtained in 2019. In 2017, the N+R+D at FR reduced GERPU by 84, and 64% at RR. Herbicides at RR with TEST adjuvants control GERPU better than without them but lower than after FR. In 2018, the efficacy was 94 (+TEST-2), 97 (+TEST-1), and 100% for other treatments. In 2019, better efficacy of N+R+D with TEST adjuvants was also found, especially TEST-2, compared to RR. It was observed that the addition of MSO and S allowed us to reduce weeds by 90, compared to 94% of N+R+D at FR. POLCO was most effectively controlled in 2019, 94–96% with TEST, and 89–98% with MSO and S. Efficacy was lower in 2017, but there was a favourable effect of all TEST adjuvants as well as the standard adjuvants on efficacy. N+R+D at FR reduced VIOAR by 74, and at RR by 67%; with TEST-1 and TEST-3, it was reduced by 78 and 72%, and 61–64% with MSO and S. In 2018 and 2019, VIOAR plants were effectively controlled regardless of the N+R+D rate and adjuvants—97–100%. A lower efficacy in 2018 was only recorded when N+R+D was applied with S—91%.

**Figure 1:**
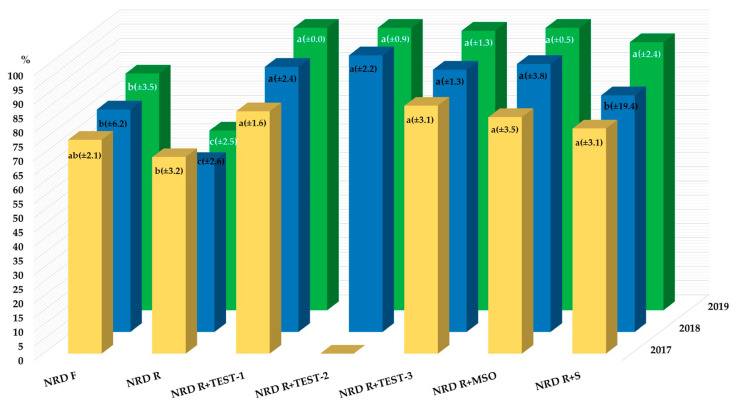
Total weed control affected by herbicides applied with adjuvants. N—nicosulfuron; R—rimsulfuron; D—dicamba; F—full rate (32.2 + 8.05 + 192.5 g L^–1^); R—reduced rate (20.0 + 5.75 + 137.5 g L^–1^); TEST-2—introduced for research in 2018; TEST-1 and TEST-2—rapeseed oil fatty acid methyl esters with surfactants, buffering agents, and anti-drift agents, differing in the amount of components in the formulations; TEST-3—surfactants with a sequestrant, humectant, and pH buffer; and standard adjuvants MSO—rapeseed oil fatty methyl esters with surfactants and a pH buffer and S—ethoxylated isodecyl alcohol. Means followed by the same letter in the column are not significantly different using Tukey’s test at *p* = 0.05 (standard deviation).

A reduction in the herbicide rate always resulted in lower efficacy, but the addition of any TEST adjuvant increased efficacy to a level significantly higher than at RR or even FR applied alone. The N+R+D with TEST adjuvants performed similarly to MSO, clearly better than with the S in 2018. In the first and third year of this study, the effectiveness of N+R+D with S though was lower than with MSO, but these differences were not statistically significant (Figure 1).

Weed control had an effect on maize grain yield and 1000 kernel weight, TKW (Table 3). The lowest yields were obtained from the untreated check in 2017 and 2019, and in 2018, they were statistically similar to yields from N+R+D at RR and FR. In 2017, the highest yields were obtained from N+R+D at FR and RR with adjuvants (11.7–12.5 t ha^–1^), significantly higher than the untreated check and N+R+D at RR—9.3 t ha^–1^. In 2018 and 2019, the highest yields were collected from N+R+D with all TEST and standard adjuvants, significantly higher than after the application of N+R+D at FR and RR without adjuvants.

The lowest TKW was found in 2017 when N+R+D at RR was applied, significantly lower than from RR + TEST-1, TEST-3, and S, but similar to the untreated check, FR, and MSO treatments (Table 3). In 2018, the least filled grain was harvested from the untreated check and N+R+D applied without adjuvants, and the most filled when TEST-2 and S adjuvants were added, although they were similar to TEST-1, TEST-3, and MSO. In 2019, the highest TKW was found when N+R+D with adjuvants was applied, higher than without adjuvants. Maize grain from the untreated check had the lowest TKW.

Based on the test results on the effects of adjuvants on living organisms (algae, daphnia, fish, honeybee) shown in Table 4 (testing by a specialised, accredited laboratory), it was concluded that the experimental adjuvants TEST-2 and TEST-3 are not classified as hazardous to the environment, with nearly 73 and more than 88% degradation after 28 days.

## 3. Discussion

Maize is a thermophilic plant with high water requirements. During the growing season, maize needs rainfall of 200 mm, and it is sensitive to water deficiency but also overflow adversely affects maize grain yield, especially when the average air temperature does not exceed 14 °C [30]. The rainfall during the growing season varied between years, covering the maize’s water needs especially in 2017. In the second and third years of this study, the rainfall was lower, yet still sufficient for a satisfactory result. At REC Brody, the average air temperature during the growing season exceeded 15 °C. According to study [31], maize yield is more dependent on the sum of precipitation than the average air temperature. At REC Brody, favourable conditions for maize development were only observed during the initial year of this study, whereas they were significantly less favourable in subsequent years. Weather conditions affect the physiological processes of target weeds as well as the efficacy of herbicides. Warmer temperatures usually enhance herbicide activity. The optimal temperature for postemergence applied herbicides is 18–29 °C. The humidity level, both high (above 70%, reduces droplet evaporation) and low (under 40%, accelerates evaporation), reduces herbicide effectiveness. Data from our weather station indicate that air humidity (60–70%) was favourable for herbicide activity. The air temperature was near-optimal in 2018 and slightly less than optimal in the other years, although it was sufficient for the effective herbicide action.

In Poland, weed communities in maize include primarily *Ch. album*, *E. crus-galli*, and other species, mainly *Polygonum* ssp., *Solanum nigrum* L., *Amaranthus retroflexus* L., and *Anthemis arvensis* L. [32]. Knowledge about weed community structure is critical for planning an effective weed management system [33]. A comparison of weed communities across the study years in REC Brody using the Sorensen index shows little similarity in weed composition, particularly between 2017 and 2018, and 2017 and 2019, but more between 2018 and 2019. During our own study, the presence of *E. crus-galli*, *Ch. album*, *G. pusillum*, *F. convolvulus*, *and V. arvensis* was found, the presence of which was also indicated in other studies [34].

In contrast to dicamba, whose solubility is not affected by the pH, sulfonylurea herbicides are dependent on the pH of spray liquid [35], and they need adjuvants that contain a surfactant or oil and a buffer solution. The results of our own study indicate a favourable effect of the tested adjuvants, i.e., TEST-2 and TEST-3, on N+R+D efficacy. This effect was particularly apparent when low-pH water (pH 4) was used as a spray liquid, practically at the level of the treatments that were prepared in pH 7 and 9 of liquid.

Great efficacy is achieved using the full herbicide rates, although, especially under favourable conditions, the same effect can also be achieved by reduced herbicide rates, especially when appropriate, comprehensive action adjuvants are added [26]. Our own research supports that herbicides perform better in the presence of adjuvants that include several substances with different modes of action. The efficacy of herbicides containing an adjuvant solely based on a single ingredient was comparable or lower, but with variations that were not always statistically substantiated. The TEST adjuvants generally affected the action of N+R+D favourably, and often even more effectively than the standard MSO adjuvant and similar or even slightly better than only the surfactant (S). A multicomponent adjuvant properly selected for a given herbicide offers the possibility of reducing the application rate of a pesticide while maintaining its high efficacy [36], which was also confirmed by our own study. It should be noted that the TEST adjuvants differed in their composition in terms of the type and amount of surfactants (TEST-1 and -2), and the TEST-3 adjuvant developed specifically for use with sulfonylurea herbicides. Newly developed adjuvant formulations allowed herbicide performance to be maintained at levels comparable to or higher than standard adjuvants. They also contain ingredients that are more environmentally friendly, so even if they were to be more expensive and just as effective as existing solutions, they are still an interesting alternative to agricultural practice.

To achieve the highest yield in maize, it is important to reduce competition from weeds. The reduction in maize yield during this study resulting from weeds’ presence was mainly determined by weather conditions, which in turn affected the growth and development of both maize and weeds. Weed species reduced maize grain yield in 2017, 2018, and 2019 by 35–52%, 35–70%, and 81–88% (control-to-herbicide-treatment comparisons). Thus, the use of herbicides makes it possible to obtain a grain yield at least 33 and even close to 90% higher than from fields without weed control [2,37].

## 4. Materials and Methods

Greenhouse experiment. *Echinochloa crus-galli* (L.) P. Beauv. (ECHCG), one of the most common weed species in maize that is easily maintained under greenhouse conditions, was chosen as the test plant. ECHCG seeds were planted in the greenhouse in plastic pots (6 cm depth, 5.0 cm diameter; 5 seedlings per pot) filled up with a mixture of soil and peat (1:1 ratio). The moisture level was maintained at 65–75%. The temperature was 20 to 25 (day) and 20 ± 2 °C (night), and the relative humidity was 60–80%. Natural sunlight was supplemented with lamps for an intensity of 600 µE m^–2^ s^–1^.

In the greenhouse, the mixture of nicosulfuron + rimsulfuron + dicamba (N+R+D, Hector Max 66.5 WG, 92 + 23 + 550 g L^–1^, DuPont, Poland) was used at full, recommended by the manufacturer (FR, 32.2 + 8.05 + 192.5 g L^–1^), and reduced rates (RR, 20.0 + 5.75 + 137.5 g L^–1^). Mixtures were applied at 2–3 leaf stages of ECHCG using a sprayer calibrated to deliver 200 L ha^–1^ through a DGTJ60 11003 VS nozzle at pressure 0.3 MPa.

The water with the desired pH (4, 7, and 9) for the preparation of the spray liquid was prepared by incorporating citric acid or K_3_PO_4_ to either lower or raise the pH of the water. N+R+D were applied alone or with tested adjuvants (all at 1.5 L^–1^): TEST-1—rapeseed oil fatty acid methyl esters with surfactants, buffering agents, and anti-drift agents; TEST-2—rapeseed oil fatty acid methyl esters with surfactants, buffering agents, and anti-drift agents; TEST-3—surfactants with a sequestrant, humectant, and pH buffer; and standard adjuvants MSO—rapeseed oil fatty methyl esters with surfactants and a pH buffer (Atpolan Bio 80 EC, 1.5 L ha^–1^, rapeseed oil methyl esters of fatty acids with surfactants and a pH buffer, Agromix, Niepołomice, Poland) and S—ethoxylated isodecyl alcohol (Trend 90 EC, 0.1% *v*/*v*, ethoxylated isodecyl alcohol, DuPont, Paris, France). Adjuvant S is an additive recommended by the manufacturer for the herbicide used in the experiment. In the experiment, adjuvant S was used as a standard solution. Adjuvants TEST-1, TEST-2, and TEST-3 were applied at 1.5 L ha^–1^, and standard adjuvants S at 0.1% and MSO at 1.5 L ha^–1^.

Tested substances are multifunctional adjuvants in the form of a concentrate for preparing a water-in-oil emulsion for use with foliar herbicides. They are oil formulations with an incorporated emulsifying and pH-buffering system for the spray liquid, and vary in quality and quantity. They differ in the type and quantity of surfactants contained in the formulation. Their proportion in the formulation also differs (trade secret of the company). The trial was designed as a randomized complete block with 3 replications repeated twice. Three weeks after application, weed control efficacy based on fresh weights of ECHCG plant reduction was determined.

Field experiment. Studies were conducted in 2017–2019 in Research and Education Center Brody (REC Brody) to evaluate the effects of TEST adjuvants on efficacy of N+R+D applied in maize. Experimental fields were located in Brody (52°43′ N, 16°29′ E), about 55 km west of Poznan, Poland. The experiment was arranged in a randomized complete block design with 4 replications. Each plot contained 4 rows spaced 70 cm apart with a plot length of 9 m. Soil was loamy sand, in respective years with pH 6.6; 5.9; and 6.3, and 1.2–1.4% of organic matter. Maize cultivar PR39H32 was sown 4 cm deep using a Monosem driller on 6 May 2017, 15 April 2018, and 24 April 2019 at 80,000 seeds/ha, harvested on 4, 10, and 29 September.

N+R+D were applied at FR (32.2 + 8.05 + 192.5 g L^–1^) and RR (20.0 + 5.75 + 137.5 g L^–1^) at 14–15 BBCH of maize with tested adjuvants TEST-1 and TEST-2 (introduced for research in 2018) and TEST-3 at 1.5 L ha^–1^, and standard adjuvants S at 0.1% and MSO at 1.5 L ha^–1^. Mixtures were applied with a wheelbarrow CO_2_-pressurized sprayer with TeeJet XR 11015 VS flat fan nozzle tips calibrated to deliver 230 L ha^–1^ at 0.22 MPa. Weeds were collected 6 weeks after application from each plot, from 2 randomly selected areas, 0.7 m^−2^. The weeds were removed from the soil surface, separated into individual species, counted, and weighed.

The resulting data (fresh weight) were used to calculate the weed management index (WCE) according to formula [38]: WCE = [(W_c_ − W_t_)/W_c_] × 100, where W_c_ is the weed fresh weight in the control plot, and W_t_ is the weed fresh weight in the treated plot. To compare weed communities between study years, the Sorensen coefficient was used, based on the weed species occurring in individual study years. The Sorensen community similarity coefficient was calculated from the formula Ss = (2a/(2a + b + c)) × 100, where a is the number of species common to both samples, b is the number of species in the first sample, and c is the number of species in the second sample [39]. The Ss index ranges in values: >80%—the species composition of groupings is almost identical, 50–80%—clear similarity, <50—low similarity.

The data necessary to develop weather conditions during the field study were obtained from a meteorological station located close to experimental plots. Herbicides with adjuvants in the following years were applied on 1 June, 24 May, and 29 May. During application, the air temperature was 15.2, 17.2, and 12.8 °C, and the humidity was 70, 60, and 65%, without any rainfall a few days before and after application.

**Figure 2:**
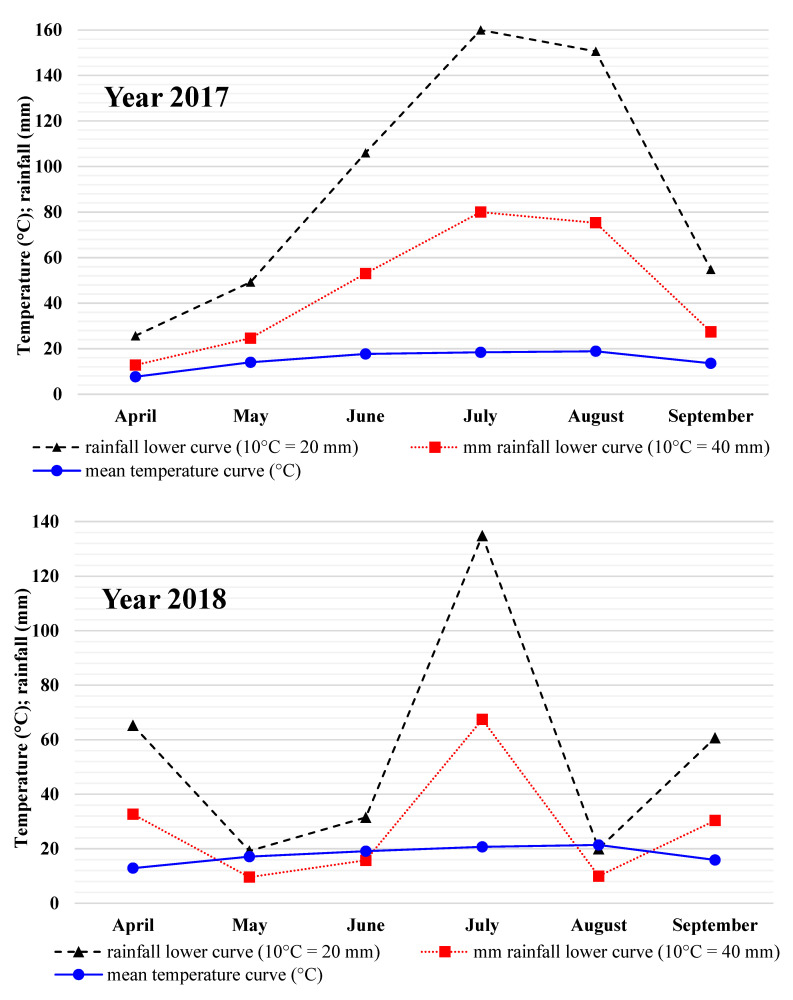

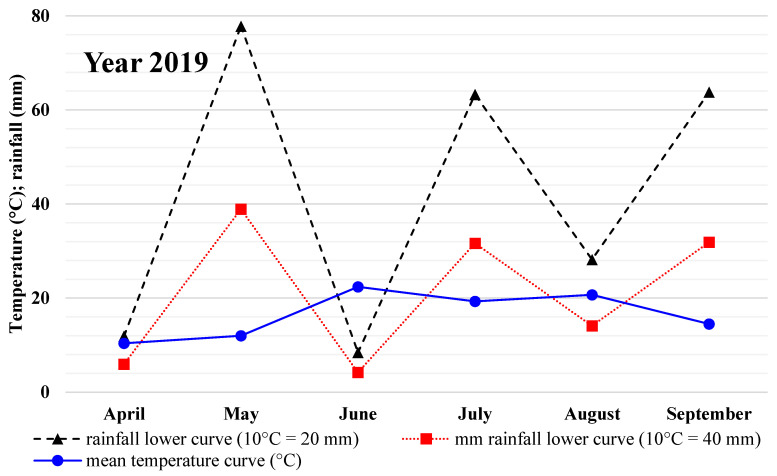
Gaussen–Walter weather diagrams for maize vegetation seasons in REC Brody.

Rainfall and thermal conditions during this study were characterized by Gaussen–Walter diagrams (Figure 2). The second, additional lowered curve of mean precipitation is the result of a modification [40] with a scale ratio of 1:4. In the diagrams, the course of the mean temperature curve between lowered rainfall curves was treated as semi-dry. The mean temperature curve above the lowered precipitation curves was described as dry.

The year 2017 was the most favourable for maize growth and development. Each month of the year experienced rainfall. In 2018, maize yield was negatively impacted by a shortage of rainfall in May, June, and August, and by excessive rainfall in April, June, and September, and in 2019 by semi-arid periods in April, June, and August (Figure 2). In successive maize growing seasons (April–September) at ZDD Brody, rainfall totals were 547.1; 331.6; and 253.4 mm.

In order to assess the effects of the selected experimental adjuvants on living organisms, samples of the adjuvants were submitted to an approved laboratory, where toxicological tests such as the algal growth inhibition test (OECD, Organisation for Economic Co-operation and Development, Guidelines for the Testing of Chemicals, test no. 201), the acute *Daphnia* immobilisation test (OECD 202), the acute fish toxicity test (OECD 203), the toxicity test for the honeybee (OECD 213 and 2014), and the degree of biodegradation were performed (OECD 301 F C.4-D (WE) 440/2008).

Statistical procedures were conducted using Statistica 13 software (StatSoft Inc., Tulsa, OK, USA). Data were subjected to ANOVA and protected Tukey’s HSD was used to separate treatment means at *p* = 0.05. Percent ratings of weed control were arc-sine-transformed prior to the analysis to correct for unequal variance. Data in tables are reported as non-transformed.

## 5. Conclusions

Multicomponent TEST adjuvants, varying in quantity and quality of components they contain, proved to be more effective than standard single-component adjuvants and comparable to or more effective than a standard multicomponent adjuvant. Properly selected multicomponent adjuvants allowed the efficacy of N+R+D to be high and stable, irrespective of the varying weather conditions during and after their application, and did not damage maize plants. The introduction of plant protection products into the market is preceded by tests, including toxicological and metabolic, as well as environmental and toxicological tests. However, tests do not pertain to ingredients of adjuvants. During this in-house study, adjuvants TEST-2 and TEST-3 were subjected to testing. The findings obtained indicate that the substances present in the aforementioned adjuvants are safe for the environment and may be utilized without any restrictions in regards to plant protection. The use of the adjuvants tested becomes even more justifiable as more importance is placed on reducing the use of chemical plant protection products in agriculture. This is undoubtedly already the case and will be even more so in the future, as the “Green Deal” project assumptions indicate.

## Figures and Tables

**Table 1:** Impact of adjuvants on efficacy of nicosulfuron + rimsulfuron + dicamba (N+R+D) mixture depending on the pH of spray liquid.

Herbicide	Rate	Adjuvant	ECHCG Control (%)			Average
			pH			
			4	7	9	
N+R+D	FR	none	48 ± 5.2 ef	48 ± 5.1 ef	56 ± 5.0 de	51 ± 3.4 D
N+R+D	RR	none	45 ± 8.3 ef	37 ± 7.1 f	42 ± 9.0 f	42 ± 6.2 E
N+R+D	RR	+TEST-1	68 ± 6.4 bc	83 ± 4.1 a	76 ± 8.1 abc	76 ± 5.3 AB
		+TEST-2	81 ± 2.4 ab	82 ± 2.4 a	80 ± 4.1 ab	81 ± 1.9 A
		+TEST-3	80 ± 1.2 ab	77 ± 11.1 ab	80 ± 2.3 ab	79 ± 4.8 A
		+MSO	73 ± 2.0 bc	81 ± 1.1 ab	79 ± 1.1 ab	78 ± 2.1 AB
		+S	66 ± 7.3 bc	71 ± 3.6 abc	63 ± 5.0 cd	67 ± 3.9 C
Average			66 ± 14.4 A	68 ± 18.2 A	68 ± 14.7 A	-

ECHCG—*E. crus-galli*; FR—full rate (32.2 + 8.05 + 192.5 g L^–1^); RR—reduced rate (20.0 + 5.75 + 137.5 g L^–1^). N+R+D—nikosulfuron + rimsulfuron + dicamba; TEST-1 and TEST-2—rapeseed oil fatty acid methyl esters with surfactants, and buffering agents and anti-drift agents, differing in the amount of components in the formulations; TEST-3—surfactants with a sequestrant, humectant, and pH buffer; and standard adjuvants MSO—rapeseed oil fatty methyl esters with surfactants and a pH buffer and S—ethoxylated isodecyl alcohol. Data are the mean of four replicates ± standard deviation. Means followed by the same letter in the column are not significantly different using Tukey’s test at *p* = 0.05.

**Table 2:** Impact of adjuvants on weed control efficacy (WCE) in the years 2017–2019.

Year	Herbicide	Rate	Adjuvant	CHEAL	ECHCG	GERPU	POLCO	VIOAR
				WCE (%)				
2017	Untreated check (g m^–2^)			3638	143	2258	572	87
	N+R+D ^1^	FR	-	84 ± 2.9 b	100 ± 0.0 a	84 ± 3.9 a	89 ± 3.5 a	74 ± 3.2 a
	N+R+D	RR	-	65 ± 6.9 c	95 ± 2.9 a	64 ± 1.7 b	83 ± 1.3 a	67 ± 7.1 a
	N+R+D	RR	+TEST-1	92 ± 3.9 a	100 ± 0.0 a	74 ± 3.4 ab	91 ± 1.8 a	78 ± 2.4 a
			+TEST-2 ^2^	-	-	-	-	-
			+TEST-3	95 ± 1.1 a	100 ± 0.0 a	73 ± 3.0 ab	90 ± 0.8 a	72 ± 4.6 a
			+MSO	95 ± 1.9 a	100 ± 0.0 a	71 ± 3.8 ab	86 ± 1.4 a	64 ± 2.5 a
			+S	82 ± 3.8 b	100 ± 0.0 a	68 ± 2.4 ab	85 ± 3.3 a	615.4 a
2018	Untreated check (g m^–2^)			1066	253	11	31	28
	N+R+D ^1^	FR	-	81 ± 6.7 b	81 ± 7.1 b	100 ± 0.0 a	86 ± 7.6 a	100 ± 0.0 a
	N+R+D	RR	-	66 ± 12.2 c	61 ± 4.5 c	100 ± 0.0 a	80 ± 7.7 a	97 ± 3.7 a
	N+R+D	RR	+TEST-1	93 ± 3.4 a	99 ± 0.7 a	97 ± 6.5 a	81 ± 5.4 a	98 ± 2.4 a
			+TEST-2 ^2^	96 ± 2.2 a	98 ± 1.0 a	94 ± 6.9 a	92 ± 5.6 a	100 ± 0.0 a
			+TEST-3	90 ± 1.8 ab	100 ± 0.0 a	100 ± 0.0 a	86 ± 5.1 a	100 ± 0.0 a
			+MSO	94 ± 3.4 a	99 ± 1.1 a	100 ± 0.0 a	81 ± 2.1 a	100 ± 0.0 a
			+S	79 ± 8.8 b	93 ± 3.4 a	97 ± 6.4 a	77 ± 5.3 a	91 ± 6.7 b
2019	Untreated check (g m^–2^)			1746	171	12	37	10
	N+R+D ^1^	FR	-	86 ± 3.2 b	85 ± 8.8 ab	94 ± 6.6 ab	100 ± 0.0 a	100 ± 0.0 a
	N+R+D	RR	-	60 ± 11.2 c	77 ± 7.5 b	67 ± 5.8 c	85 ± 3.8 b	97 ± 6.8 a
	N+R+D	RR	+TEST-1	99 ± 1.4 a	96 ± 2.3 a	87 ± 5.5 b	96 ± 5.3 a	98 ± 4.1 a
			+TEST-2 ^2^	99 ± 0.9 a	90 ± 8.1 ab	100 ± 0.0 a	94 ± 6.6 a	100 ± 0.0 a
			+TEST-3	99 ± 1.1 a	90 ± 2.0 ab	86 ± 4.2 b	96 ± 8.1 a	100 ± 0.0 a
			+MSO	99 ± 0.9 a	94 ± 5.5 a	90 ± 11.7 ab	98 ± 2.1 a	100 ± 0.0 a
			+S	96 ± 2.9 a	88 ± 11.5 ab	90 ± 3.7 ab	89 ± 15.8 ab	100 ± 0.0 a

^1^ N—nicosulfuron; R—rimsulfuron; D—dicamba; FR—full rate (32.2 + 8.05 + 192.5 g L^–1^); RR—reduced rate (20.0 + 5.75 + 137.5 g L^–1^); ^2^ TEST-2 introduced in 2018; CHEAL—*Ch. album*; ECHCG—*E. crus-galli*; GERPU—*G. pusillum*; POLCO—*F. convolvulus*; VIOAR—*V. arvensis.* TEST-1 and TEST-2—rapeseed oil fatty acid methyl esters with surfactants, buffering agents, and anti-drift agents, differing in the amount of components in the formulations; TEST-3—surfactants with a sequestrant, humectant, and pH buffer; and standard adjuvants MSO—rapeseed oil fatty methyl esters with surfactants and a pH buffer and S—ethoxylated isodecyl alcohol. Data are the mean of four replicates ± standard deviation. Means followed by the same letter in the column are not significantly different using Tukey’s test at *p* = 0.05.

**Table 3:** Impact of weed control on grain yield and thousand kernel weight (TKW).

Herbicide	Rate	Adjuvant	Grain Yield (t ha^–1^)			TKW (g)		
			2017	2018	2019	2017	2018	2019
Untreated check	-	-	6.0 ± 1.58 c	2.5 ± 0.86 b	2.2 ± 1.05 c	276 ± 20.5 ab	238 ± 11.1 c	248 ± 22.8 c
N+R+D	FR	none	11.7 ± 0.45 a	4.2 ± 0.16 b	7.1 ± 1.13 b	296 ± 14.6 ab	247 ± 12.2 bc	287 ± 18.7 b
N+R+D	RR	none	9.3 ± 0.91 b	3.9 ± 0.86 b	7.1 ± 0.92 b	261 ± 12.6 b	245 ± 16.2 bc	271 ± 17.5 b
N+R+D	RR	+TEST-1	12.5 ± 0.22 a	8.3 ± 1.64 a	10.7 ± 1.05 a	306 ± 10.2 a	270 ± 14.7 ab	321 ± 6.1 a
		+TEST-2	-	7.8 ± 2.00 a	9.2 ± 0.71 ab	-	289 ± 19.0 a	324 ± 16.4 a
		+TEST-3	12.4 ± 0.21 a	7.4 ± 2.34 a	10.4 ± 1.52 a	308 ± 10.3 a	276 ± 8.0 ab	314 ± 7.3 a
		+MSO	11.7 ± 0.21 a	7.6 ± 1.26 a	10.9 ± 0.80 a	290 ± 13.3 ab	275 ± 14.3 ab	325 ± 10.0 a
		+S	12.0 ± 0.39 a	6.6 ± 1.25 a	11.1 ± 0.92 a	298 ± 14.7 a	283 ± 10.8 a	320 ± 5.9 a

FR—full rate; RR—reduced rate; N—nicosulfuron; R—rimsulfuron; D—dicamba; FR—full rate (32.2 + 8.05 + 192.5 g L^–1^); RR—reduced rate (20.0 + 5.75 + 137.5 g L^–1^); TEST-2—introduced for research in 2018; TEST-1 and TEST-2—rapeseed oil fatty acid methyl esters with surfactants, buffering agents, and anti-drift agents, differing in the amount of components in the formulations; TEST-3—surfactants with a sequestrant, humectant, and pH buffer; and standard adjuvants MSO—rapeseed oil fatty methyl esters with surfactants and a pH buffer and S—ethoxylated isodecyl alcohol. Data are the mean of four replicates ± standard deviation. Means followed by the same letter in the column are not significantly different using Tukey’s test at *p* = 0.05.

**Table 4:** Effect of experimental adjuvants on living organisms.

Type of Test	TEST-2	TEST-3
IC50 (72 h)—OECD 201 ^1^	3.157 mg L^–1^	˃100 mg L^–1^
LOEC ^1^ (72 h)	2.92 mg L^–1^	-
NOEC ^1^ (72 h)	1.47 mg L^–1^	-
EC50 (48 h)—OECD 202 ^2^	˃100 mg L^–1^	˃100 mg L^–1^
LC50 (96 h)—OECD 203 ^3^	˃100 mg L^–1^	˃100 mg L^–1^
NOED—OECD 213 ^4^	˃100 µg L^–1^	˃100 µg L^–1^
NOED—OECD 214 ^4^	˃100 µg L^–1^	˃100 µg L^–1^
SB—OECD 301	72.9 ± 2.2	88.1 ± 2.9

^1^ The growth inhibition test on the alga *Pseudokirchneriella subcapitata*; ^2^ the acute immobilisation test on the *Daphnia magna*; ^3^ the acute toxicity test on the fish, Danio rerio—striped danios; ^4^ the acute oral toxicity test on the honeybee; SB—degree of biodegradation after 28 days, F C.4-D (EC) 440/2008. TEST-2—rapeseed oil fatty acid methyl esters with surfactants, buffering agents, and anti-drift agents; TEST-3—surfactants with a sequestrant, humectant, and pH buffer.

## Data Availability

Available upon reasonable request.
